# Racial residential segregation is associated with ambient air pollution exposure after adjustment for multilevel sociodemographic factors: Evidence from eight US-based cohorts

**DOI:** 10.1097/EE9.0000000000000367

**Published:** 2025-01-20

**Authors:** Hiwot Y. Zewdie, Carolyn A. Fahey, Anna L. Harrington, Jaime E. Hart, Mary L. Biggs, Leslie A. McClure, Eric A. Whitsel, Joel D. Kaufman, Anjum Hajat

**Affiliations:** a Department of Epidemiology, University of Washington School of Public Health, Seattle, Washington; b Department of Environmental Health, Harvard T.H. Chan School of Public Health, Boston, Massachusetts; c Department of Biostatistics, University of Washington School of Public Health, Seattle, Washington; d College for Public Health and Social Justice, Saint Louis University, St. Louis, Missouri; e Department of Epidemiology, Gillings School of Global Public Health, University of North Carolina, Chapel Hill, North Carolina; f Department of Medicine, School of Medicine, University of North Carolina, Chapel Hill, North Carolina; g Department of Environmental and Occupational Health, University of Washington School of Public Health, Seattle, Washington; h Department of Medicine, University of Washington School of Medicine, Seattle, Washington

**Keywords:** Racial residential segregation, Socioeconomic factors, Air pollution

## Abstract

**Objective:**

We examined if racial residential segregation (RRS) – a fundamental cause of disease – is independently associated with air pollution after accounting for other neighborhood and individual-level sociodemographic factors, to better understand its potential role as a confounder of air pollution-health studies.

**Methods:**

We compiled data from eight large cohorts, restricting to non-Hispanic Black and White urban-residing participants observed at least once between 1999 and 2005. We used 2000 decennial census data to derive a spatial RRS measure (divergence index) and neighborhood socioeconomic status (NSES) index for participants’ residing Census tracts, in addition to participant baseline data, to examine associations between RRS and sociodemographic factors (NSES, education, race) and residential exposure to spatiotemporal model-predicted PM_2.5_ and NO_2_ levels. We fit random-effects meta-analysis models to pool estimates across adjusted cohort-specific multilevel models.

**Results:**

Analytic sample included eligible participants in CHS (N = 3,605), MESA (4,785), REGARDS (22,649), NHS (90,415), NHSII (91,654), HPFS (32,625), WHI-OS (77,680), and WHI-CT (56,639). In adjusted univariate models, a quartile higher RRS was associated with 3.73% higher PM_2.5_ exposure (95% CI: 2.14%, 5.32%), and an 11.53% higher (95% CI: 10.83%, 12.22%) NO_2_ exposure on average. In fully adjusted models, higher RRS was associated with 3.25% higher PM_2.5_ exposure (95% CI: 1.45%, 5.05%; *P* < 0.05) and 10.22% higher NO_2_ exposure (95% CI: 6.69%, 13.74%; *P* < 0.001) on average.

**Conclusions:**

Our findings indicate that RRS is associated with the differential distribution of poor air quality independent of NSES or individual race, suggesting it may be a relevant confounder to be considered in future air pollution epidemiology studies.

**What this study adds:**

Few studies have considered racial residential segregation (RRS) in air pollution epidemiology. It is unlikely that the association between RRS, as a manifestation of structural racism, and environmental health risk, is adequately accounted for by other contextual-level sociodemographic factors. Our findings show that RRS is associated with the differential distribution of poor air quality independent of neighborhood socioeconomic status or individual race implying that it could be an important confounder in air pollution epidemiology studies. Our work explores one-way structural racism can operate to shape environmental health risk beyond what is typically considered in environmental health studies; we urge researchers to critically consider the historical contexts that shape environmental and health risk to appropriately quantify air pollution-health effects.

## Introduction

Exposure to ambient air pollution is linked to a range of poor health outcomes across the life course,^1^ including cardiovascular disease,^2–4^ asthma,^5,6^ cancer,^4,7^ and dementia.^8,9^ In the United States (US), exposure to environmental hazards is disproportionately distributed across racial and socioeconomic groups.^10–16^ A study examining national air pollution trends from 1990 to 2010 found racial and ethnic minority groups were consistently exposed to higher levels of air pollutants (fine particulate matter [PM_2.5_], nitrogen dioxide [NO_2_], ozone [O_3_], sulfur dioxide [SO_2_], particulate matter [PM_10_], and carbon monoxide [CO]) over time.^10^ Similarly, national mean exposures were highest for lowest income households compared with highest income households.^10^ The well-established racial and socioeconomic status (SES) disparities in most health outcomes suggest race and SES are important confounders in air pollution epidemiology research.

Environmental health researchers have become more diligent in accounting for social determinants in efforts to accurately quantify the effects of air pollution on health. Namely, the potential for confounding by SES is well recognized, resulting in thorough adjustment of multidimensional, individual, and area-level measures of SES.^17,18^ More recently, racial residential segregation (RRS) has emerged as a potentially important factor in air pollution epidemiology studies.

RRS – a form of structural racism – is the degree to which different racial groups reside separately from each other.^19,20^ In the US, residential patterning of racial and ethnic groups has been shaped through historical and racialized housing policies,^21–23^ economic institutions,^24–26^ and legislative systems,^25,27^ and has been sustained through continued discriminatory housing practices even after the dissolution of legalized segregation.^21,28–31^ Historical zoning practices continue to contribute to the deterioration of neighborhood quality in racialized communities, maintaining unequal residential patterns, and resulting in the differential distribution of health-promoting resources – quality education, public amenities, and healthcare – on the basis of race. Neighborhood attainment research demonstrates that the legacy of these racialized practices supersedes individual-level characteristics, like SES, such that even the highest earning racial minority individuals are less likely than White individuals with equivalent SES to reside in neighborhoods with an area-level SES commensurate with their own.^32–34^ In other words, RRS operates to shape access to resources for racially marginalized communities through mechanisms independent of SES. As such, RRS is regarded as a fundamental cause of racial disparities in health.^20,35–37^

RRS as a fundamental cause of health, operates by (1) driving access to SES-based resources (e.g., income, education) and (2) driving access to non-SES resources (e.g., collective power, freedom).^37,38^ More specifically, racial health disparities persist because racism is a fundamental cause of racial differences in SES, another fundamental cause of health inequalities, and because racism shapes power, prestige, and neighborhood contexts, among other factors, which also have fundamental associations with health independent of SES.^37^

**Figure 1.**
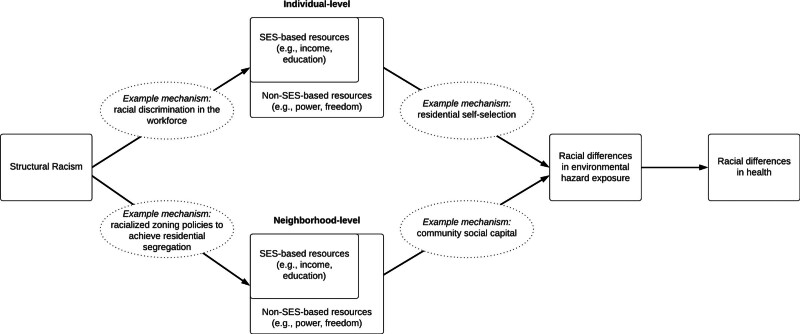
Conceptual model: structural racism as a fundamental cause of racial differences in environmental hazard exposure.

Under a fundamental cause framing, we can understand how differential environmental health risk across segregated communities can result through both SES and non-SES mechanisms. In Figure 1, we depict how these relationships across the individual and neighborhood levels may lead to racial differences in environmental hazard exposure and subsequent racial health disparities. It is unlikely that structural factors that drive environmental health disparities are sufficiently adjusted away by the inclusion of intermediary, and largely SES-based sociodemographic factors, that are commonly used in air pollution research (proposed directed acyclic graph in Supplemental Material 1; http://links.lww.com/EE/A323).

The established association between RRS and several health outcomes,^20,35–37,39^ and the potential association between RRS and air pollution that remains after accounting for SES-based variables in air pollution epidemiology research, suggest there may be residual confounding by structural racism in air pollution-health research. To investigate the latter, we leveraged data from eight large cardiovascular disease cohorts to (1) assess the association between RRS and ambient air pollution and (2) examine if RRS is independently associated with air pollution after accounting for neighborhood and individual-level sociodemographic factors commonly used in air pollution research.

## Methods

This study used data from eight cardiovascular disease cohorts (Table 1), including the Cardiovascular Health Study (CHS), Multi-Ethnic Study of Atherosclerosis (MESA) and MESA Air, Reasons for Geographic And Racial Differences in Stroke (REGARDS), Nurses’ Health Study (NHS), Nurses’ Health Study II (NHSII), Health Professionals Follow-up Study (HPFS), Women’s Health Initiative – Observational Study (WHI-OS), and the Women’s Health Initiative – Clinical Trials (WHI-CT). All cohorts collected baseline data, including sociodemographic factors and address history, and included follow-up health outcome data. Sociodemographic variables were harmonized across cohorts to facilitate comparable analyses.

**Table 1.** Cohort characteristics

Cohort	Enrollment	Age	Sex	Location
Nurses’ Health Study (NHS)	1976	30 to 55	Female	Continental US
Nurses’ Health Study II (NHSII)	1989	25 to 42	Female	Continental US
Health Professionals’ Follow-up Study (HPFS)	1986	40 to 75	Male	Continental US
Women’s Health Initiative – Observational Study (WHI-OS)	1993 to 1998	50 to 79	Female	40 Centers across US
Women’s Health Initiative – Clinical Trial (WHI-CT)	1993 to 1998	50 to 79	Female	40 Centers across US
Cardiovascular Health Study (CHS)	1989 to 1993	65+	Female, male	4 US Sites
Multi-Ethnic Study of Atherosclerosis (MESA)	2000 to 2002	45 to 84	Female, male	6 US Sites
Reasons for Geographic and Racial Differences in Stroke (REGARDS)	2003 to 2007	45+	Female, male	Continental US, focus on Southeast

We defined 1999 to 2005 as the time period of interest for this analysis to capture the first observation after enrollment where both PM_2.5_ and NO_2_ were available and within 5 years of exposure measurement (2000). Participants without at least one observation over the course of follow-up were excluded from the analytic sample. The analytic sample was also limited to non-Hispanic Black and non-Hispanic White participants due to the limited sample sizes of other racial and ethnic groups. We further limited the analytic sample to participants living in urban areas because of differences in segregation patterns in rural versus urban areas.^40^ As such, we only included participants living in a Census tract with a rural-urban commuting code (RUCA) of 1 to 3.^41^ Participants missing exposure, outcome, or covariate data also were excluded from the analytic sample (details included in Supplemental Material 2; http://links.lww.com/EE/A323). The study protocol for this analysis was approved by the institutional review board at the University of Washington and by all participating cohorts.

### Exposures

#### Individual-level variables

Race and ethnicity and educational attainment at baseline were included as individual-level primary exposures. Participants were categorized as non-Hispanic Black or non-Hispanic White based on self-reported race and ethnicity.(Supplemental Material 3; http://links.lww.com/EE/A323) Race and ethnicity were included to proxy for other levels of racism (e.g., interpersonal).^39^ Self-reported educational attainment was dichotomized as less than or greater than or equal to high school.

#### Neighborhood-level variables

Measures of RRS and neighborhood socioeconomic status (NSES) were derived for each Census tract in the US using 2000 Decennial Census data and linked to participants’ residing tract for their observation closest to 2000.

Racial residential segregation was measured using the divergence index,^42^ a multirace segregation measure indicating the magnitude of which the racial and ethnic composition of a smaller geographic unit (i.e., Census tract) is divergent from the racial and ethnic composition of a larger geographic unit (i.e., core-based statistical area [CBSA] or county for those who do not reside in a CBSA). Acknowledging residential segregation as a spatial phenomenon, we developed a spatial version of the measure that incorporates the racial and ethnic composition of neighboring tracts.^43,44^ Spatializing the measure allows us to overcome the “checkerboard” problem inherent to nonspatial segregation measures and create a segregation measure that better aligns with its conceptualization.^43,44^ The spatial divergence index was calculated as follows:


$$\begin{array}{l} D_{spatial\;tract_{i}\;}=\;\overset{J_{groups}}{\underset{j\;=1}{\sum }}p_{\left(i+s\right)\;j}*ln\frac{p_{\left(i+s\right)\;j}}{P_{j}} \end{array}$$

where J is the total number of racial and ethnic groups, $P_{j}$ is the proportion of group j out of the total population of the CBSA, $p_{\left(i+s\right)\;j\;}$ is the proportion of group j in each tract i and its spatial neighbors s, with the counts of neighboring tracts weighted using an inverse distance function. Spatial divergence was calculated for all Census tracts in the US, regardless of urbanicity, and then transformed into a percentile measure to rank each Census tract from lowest to highest segregation (0 to 1) (Distribution of national spatial divergence rank values for participants residing in urban Census tracts is depicted in Supplemental Material 5; http://links.lww.com/EE/A323).

#### Neighborhood socioeconomic status index

We used a compositional measure of NSES developed by Christine et al^45^ for all Census tracts in the US (Supplemental Material 4; http://links.lww.com/EE/A323). This is a measure that has been used extensively in air pollution research.^46–50^ To create a cross-sectional version of the index appropriate for the present analysis, we used principal component analysis to reweight the 15 Census 2000 variables used in the Christine et al. index. These variables represent the following socioeconomic domains: education, employment, housing, income/wealth, occupation, and residential stability. Standardized Census variables were multiplied by the new principal component analysis factor weights and then mean-centered to derive a national NSES index for this study. Higher scores on the index indicate higher socioeconomic disadvantage.

### Outcomes

#### Ambient air pollution

Residential exposure to PM_2.5_ and NO_2_ (a traffic-related air pollutant) was estimated using a national spatiotemporal model. This model is an extension of a universal kriging model that leverages historical and contemporary patterns of pollutant concentrations, geographic characteristics, and spatial smoothing to predict daily residential exposure. Geographic characteristics include proximity measures (distance to nearest major road, intersection, truck route, railway, railyard, coastline, airport, and port) and buffer measures (major road length, truck route length, land-use category, long-term vegetation index, population density, and emission sources).^51^ Annual averaged air pollution exposure was calculated by averaging 2-week exposure estimates for the year before participants’ closest observation to 2000.^51,52^

#### Covariates

For all cohorts, harmonized sociodemographic variables including sex (male or female), baseline marital status (married or not married), and continuous age were included as covariates. For MESA, study site was included as an additional individual-level covariate. Urbanicity of the Census tract (RUCA, 1 to 3) and Census region (Northeast, Midwest, South, West) were the only variables included as area-level covariates. We chose these area-level covariates based on prior work,^53–56^ and the understanding of racial segregation as a driver of neighborhood effects.^20,36,57^

### Statistical analysis

To examine the association between individual and neighborhood sociodemographic factors and ambient air pollution, cohort-specific multilevel linear regression models adjusted for the aforementioned covariates were fit for PM_2.5_ and NO_2_ separately. Air pollution concentrations were log-transformed and interpreted as percent changes. RRS was transformed such that a one-unit difference corresponded to a quartile difference in RRS rank nationally. This transformation was chosen for standardized and readily interpretable measurement of segregation that can be used across cohorts. NSES was standardized such that a one-unit difference corresponded to a standard deviation difference from the national mean NSES. Thin plate spatial splines were constructed and included in the models to account for spatial dependence of the air pollutants and minimize spatial confounding.^58^ Additionally, a random intercept for Census tract was included.

Estimates from each cohort model were pooled using a DerSimonian-Laird (random effects)^59^ estimator yielding an average effect for each air pollutant. A random-effects meta-analytic approach was used because of challenges in data sharing across the different cohorts and for some cohort-specific variables. This approach is as efficient and not more or less biased than a one-stage, individual participant data (IPD) pooling approach.^60,61^ We harmonized data to have consistency in the measurement of covariates across cohorts. Cochran’s Q test of homogeneity, standard deviation (τ), and Higgins’ I squared were used to determine the homogeneity of effects across cohorts. A staged-modeling approach was used to examine changes in effect sizes with a staged inclusion of the primary exposures. The first set of models aimed to understand the independent effect of each primary exposure on air pollution; separate univariate models were fit between each primary exposure and both air pollutants. Subsequent models were then fit to estimate the independent effect of RRS and NSES on air pollution after accounting for its corresponding individual-level measures (i.e., model 1: RRS and race, and model 2: NSES and education). Then a single model including both RRS and NSES was fit to estimate the independent effect of RRS after adjusting for NSES. Finally, a fully adjusted model including RRS, NSES, race, and education was fit to examine the independent effect of RRS on air pollution after accounting for the other sociodemographic measures. The average pooled effect for RRS was compared across all four sets of models to understand better how race, education, and NSES influence the association between RRS and air pollution. Pooled effect estimates can be interpreted as the average percent change in air pollution concentration for a one-unit increase in the exposure of interest across all cohorts. Robust standard errors were used to construct 95% confidence intervals. All statistical analyses were performed using R version 4.2.1.

## Results

**Figure 2.**
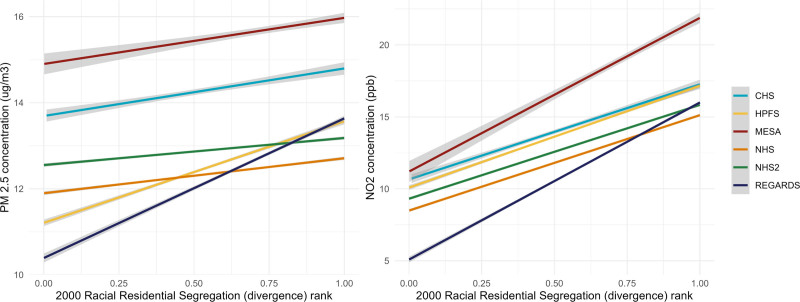
Crude association between RRS and air pollution. WHI-OS and WHI-CT excluded from figure due to data sharing limitations.

Based on our exclusion criteria, analytic sample sizes for our eight cohorts were as follows: CHS (N = 3,605), MESA (4,785), REGARDS (22,649), NHS (90,415), NHSII (91,654), HPFS (32,625), WHI-OS (77,680), and WHI-CT (56,639). Given the varying recruitment timelines and eligibility criteria of each cohort, we observe variation in several participant-level characteristics (Table 2). NHSII and MESA were the youngest cohorts with median age (interquartile range [IQR]) of 35 (32, 39) and 51 (42, 58), respectively, compared with the other cohorts. MESA and REGARDS comprised the highest proportions of non-Hispanic Black participants relative to the other cohorts (44% and 45%, respectively). CHS comprised the greatest proportion of participants without at least a high school education (27%) compared with the other cohorts. Cohorts were relatively similar across contextual features, with modest variation. Median NSES z-scores ranged from −0.72 (WHI-OS), indicating that most participants in this cohort lived in neighborhoods that had less neighborhood economic disadvantage relative to other neighborhoods in the US, to 0.47 (REGARDS), indicating most participants in this cohort lived in neighborhoods that had relatively greater neighborhood economic disadvantage. Participants across cohorts resided in neighborhoods that had relatively similar levels of RRS, except for participants in MESA and REGARDS. These cohorts had participants living in neighborhoods that ranked nationally in the upper quartile for RRS, i.e., residential Census tracts were more divergent from their surrounding CBSA (MESA: 0.79, IQR: 0.56, 0.94; REGARDS: 0.76, IQR: 0.45, 0.94). There was a similar distribution in residential air pollution exposure across all cohorts for both PM_2.5_, which ranged from 12 to 16 µg/m^3^, and NO_2_, which ranged from 11 to 16 ppb. Crude descriptions indicate a modest positive linear association between RRS and both air pollutants that is consistent across all cohorts (*P* <0.001 for all cohort-specific univariate models), although some cohorts (e.g., REGARDS) have steeper slopes indicating variability in the RRS – AP association (Figure 2).

**Table 2.** Descriptive characteristics of eight prospective cohorts

	Cohort							
	CHS	MESA	REGARDS	NHS	NHSII	HPFS	WHI-OS	WHI-CT
N	3,605	4,785	22,649	90,415	91,654	32,625	77,680	56,639
Age, median (IQR)	71 (68, 75)	51 (42, 58)	62 (56, 70)	56 (50, 63)	35 (32, 39)	57 (49, 65)	68 (62, 73)	67 (62, 72)
Non-Hispanic Black, N (%)^a^	557 (15)	2,113 (44)	10,166 (45)	1,984 (2)	1,908 (2)	375 (1)	7,124 (9)	6,497 (11)
Educational achievement < high school, N (%)^a^	961 (27)	364 (8)	2,727 (12)	0 (0)	0 (0)	0 (0)	3,162 (4)	2,515 (4)
NSES, Median (IQR), z-score	−0.18 (−0.70, 0.56)	0.14 (−0.81, 0.83)	0.47 (−0.36, 1.16)	−0.60 (−1.27, 0.01)	−0.52 (−1.12, 0.06)	−0.57 (−1.31,0.13)	−0.72 (−1.41, 0.01)	−0.58 (−1.29, 0.13)
RRS, Median (IQR), rank	0.50 (0.27, 0.67)	0.79 (0.56, 0.94)	0.76 (0.45, 0.94)	0.53 (0.34, 0.73)	0.53 (0.33, 0.72)	0.53 (0.31, 0.75)	0.57 (0.36, 0.79)	0.58 (0.36, 0.79)
PM_2.5_, Median (IQR), ug/m3	14 (13, 16)	16 (14, 17)	12 (11, 14)	12 (10, 14)	13 (11, 15)	12 (10, 14)	12 (11, 15)	13 (10, 15)
NO_2_, Median (IQR), ppb	14 (10, 18)	16 (12, 24)	11 (8, 16)	11 (7, 15)	12 (8, 15)	13 (9, 17)	13 (10, 18)	13 (10, 18)

a Characteristics measured at enrollment. All other characteristics are measured at observation closest to 2000.

**Figure 3.**
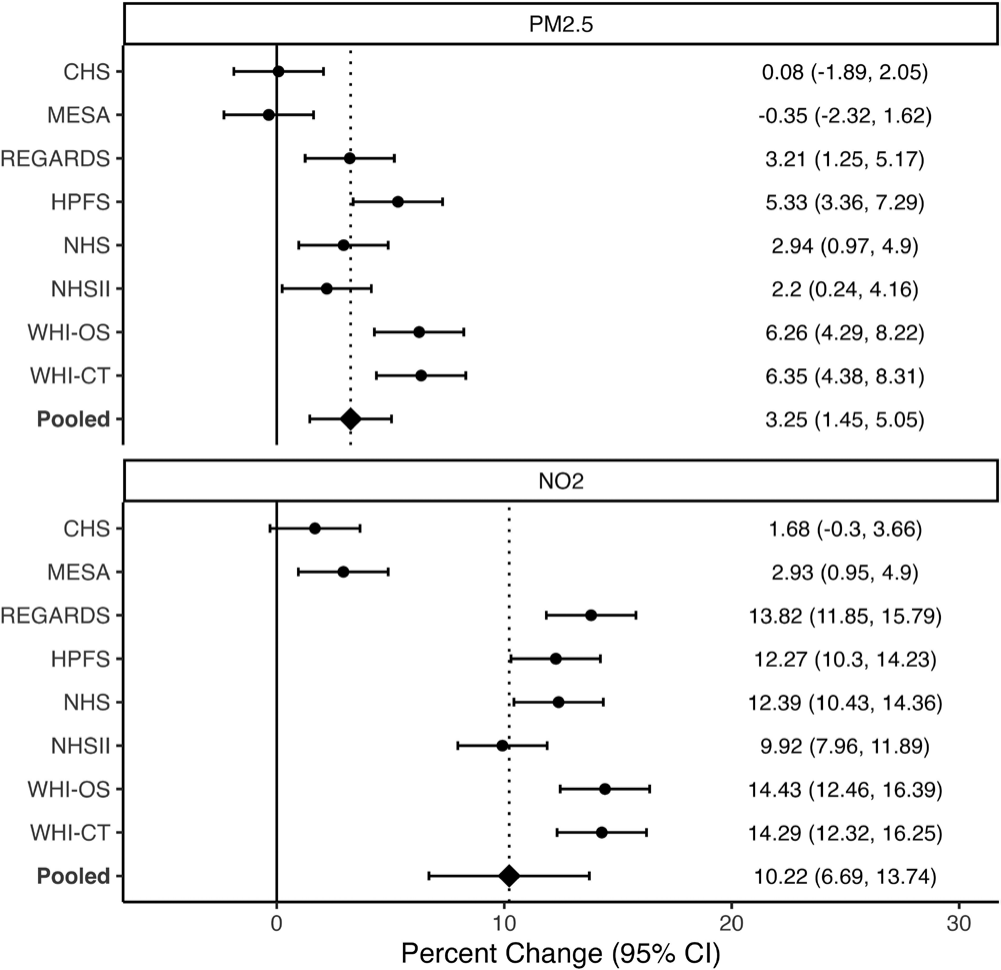
Cohort-specific results for RRS coefficient in fully adjusted models. Random-effects meta-analysis model heterogeneity statistics: PM_2.5_: *I*^2^ = 85.1% (72.4%, 91.9%), τ= 2.39 (1.39, 5.18), Q = 46.88 (*P* < 0.001); NO_2_ *I*^2^ = 96.1% (94.1%, 97.4%), τ^2^ = 4.99 (3.22, 10.32), Q = 179.68 (*P* < 0.001).

Adjusted multilevel models further support this observation (Table 3). Specifically, in adjusted univariate models, a quartile higher RRS was associated with an average pooled effect of 3.73% higher residential PM_2.5_ exposure (95% CI: 2.14%, 5.32%; *P* < 0.001), and 11.53% higher residential NO_2_ exposure (95% CI: 10.8%3, 12.22%; *P* < 0.001). This positive association remained, though slightly attenuated, after fitting subsequent models that accounted for commonly adjusted individual and neighborhood-level characteristics. In the final model adjusted for region, RUCA, sex, age, MESA site (where applicable), individual race, individual education, and NSES, a quartile increase in RRS was associated with an average pooled effect of 3.25% higher PM_2.5_ exposure (95% CI: 1.45%, 5.05%; *P* < 0.05) and 10.22% higher NO_2_ exposure (95% CI: 6.69%, 13.74%; *P* < 0.001). Forest plots displayed in Figure 3 show similar effect sizes across most cohorts, though effect sizes appear to be attenuated or null for MESA- and CHS-specific models. Tests of heterogeneity confirm statistical heterogeneity (PM_2.5_: *I*^2^ = 85.1% [72.4%, 91.9%], τ = 2.39 [1.39, 5.18], Q = 46.88 [*P* < 0.001]; and NO_2_ *I*^2^ = 96.1% [94.1%, 97.4%], τ = 4.99 [3.22, 10.32], Q = 179.68 [*P* < 0.001]).

**Table 3.** Meta-analysis results for associations between exposures and air pollution

	Univariable	Race + RRS	Education + NSES	RRS + NSES	Fully adjusted
PM_2.5_					
Individual variables					
Non-Hispanic Black race	4.54 (2.17, 6.91)	3.63 (1.60, 5.65)	-	-	2.71 (1.07, 4.36)
Less than HS	0.64 (−0.24, 1.52)^a^	-	0.41 (−0.47, 1.28)	-	0.29 (−0.59, 1.17)
Neighborhood variables					
RRS (quantile)	3.73 (2.14, 5.32)	3.45 (1.78, 5.12)	-	3.43 (1.69, 5.17)	3.25 (1.45, 5.05)
NSES (z-score)	3.41 (2.57, 4.24)	-	3.39 (2.56, 4.23)	2.88 (2.08, 3.68)	3.12 (2.38, 3.85)
NO_2_					
Individual variables					
Non-Hispanic Black race	13.85 (7.31, 20.38)	10.19 (5.23, 15.16)	-	-	8.05 (3.79, 12.30)
Less than HS	1.37 (−0.20, 2.93)^a^	-	0.77 (−0.81, 2.34)	-	0.41 (−1.27, 2.08)
Neighborhood variables					
RRS (quantile)	11.53 (10.83, 12.22)	10.69 (7.46, 13.91)	-	10.78 (7.46, 14.11)	10.22 (6.69, 13.74)
NSES (z-score)	6.95 (5.16, 8.75)	-	6.94 (5.15, 8.72)	6.02 (5.17, 6.88)	5.86 (4.94, 6.78)

Coefficients represent percent change in air pollutant for a unit increase in exposure. All models adjusted for region, RUCA, sex, age, MESA-site (where applicable). One-unit increase corresponds to White vs. Black race, greater than or equal to high school education vs. less than high school, quartile difference in RRS, standard deviation difference in NSES.

a Pooled estimate excludes NHS, NHSII, and HPFS due to the lack of variability in education for these cohorts

Of note, in the fully adjusted model, race and ethnicity and NSES also remained positively associated with air pollution exposure. Specifically, non-Hispanic Black participants were estimated to be exposed to PM_2.5_ concentrations 2.71% higher (95% CI: 1.07%, 4.36%; *P* < 0.05) and NO_2_ concentrations 8.05% higher (95% CI: 3.79%, 12.30%; *P* < 0.05) than that of their non-Hispanic White counterparts on average across cohorts. Greater neighborhood economic disadvantage was associated with a 3.12% higher PM_2.5_ exposure (95% CI: 2.38%, 3.85%; *P* < 0.001) and a 5.56% higher NO_2_ exposure (95% CI: 4.94%, 6.78%; *P* < 0.001) compared with neighborhoods experiencing less economic disadvantage (by one standard deviation) on average.

## Discussion

In this study, we used data from eight large cohorts to assess associations between RRS and residential exposure to ambient air pollutants, PM_2.5_ and NO_2_ in urban tracts. We found that higher tract-level racial segregation was associated with higher residential exposure to air pollution and that this association remained after adjusting for individual- and neighborhood-level sociodemographic variables. In addition to RRS, individual-level race and ethnicity and NSES were positively associated with air pollution exposure in fully adjusted models. In all models, we observed associations of larger magnitude when assessing relationships between sociodemographic factors and residential exposure to NO_2_ compared with PM_2.5_.

Even though legalized racial residential segregation has long been dissolved, our findings suggest that the legacy of these practices continues to have tangible impacts on the living conditions of racial and ethnic minority communities, beyond what can be attributed to their individual or NSES.^62–66^ Remnants of historical discriminatory zoning decisions continue to influence where pollutants are distributed and which communities bear the greatest burden.^67–69^ For example, the designation of “locally unwanted land uses” has been used to reinforce the distribution of high polluting sources in predominantly Black and Hispanic communities.^70^ Polluting industries exploit the time, collective power, and community organizing needed to establish protections for often already politically disenfranchised racialized communities.^71,72^ This leaves these communities disproportionally exposed to environmental hazards and the subsequent health implications, driven by mechanisms rooted in structural racism.^70,73^

Our findings are consistent with other studies examining RRS and air pollution. Woo et al^55^ examined metropolitan-level RRS and PM_2.5_, PM_10_, and NO_2_, and found that more segregation was significantly associated with higher exposure to all three air pollutants. Jones et al^53^ similarly found that living in more racially segregated neighborhoods was associated with higher exposure to PM_2.5_ and oxides of nitrogen (NO_x_ – a broader classification of nitrogen oxides that includes NO_2_). In both studies, these effects were observed even after adjusting for individual-level race and ethnicity,^53,55^ and after additionally adjusting for individual-level socioeconomic characteristics, education, and household income.^53^ Jones et al further adjusted for Census tract median household income and found that the positive association between RRS and both air pollutants (PM_2.5_ and NO_X_) remained, further corroborating our findings. Similarly, a study by Ekengaa et al found that racially segregated neighborhoods, regardless of their economic segregation levels, were more likely to be located in air-toxic hot spots compared with neighborhoods that were not racially and economically segregated. Together, these studies reinforce the presence of an independent effect of RRS on air pollution that persists even after adjustment for multilevel sociodemographic factors, suggesting RRS to be a potentially relevant confounder to be considered in air pollution-health studies.

It is important to note that the relationship between RRS, air pollution, and health is complex. For a clearer argument in this paper, we assume a unidirectional association between RRS and air pollution, such that RRS precedes air pollution. However, it is also possible for air pollution to drive and sustain RRS through postciting demographic changes.^69^ In these instances, RRS would lie on the causal pathway between air pollution and a given health outcome, making it inappropriate to adjust for. This is not to say structural racism is not at play; as a fundamental driver of health inequities, structural racism may operate in other ways to shape environmental health risk (e.g., discriminatory practices that keep racially and ethnically marginalized communities from moving out of polluted neighborhoods). Our work explores one-way structural racism can operate to shape environmental health risk; we urge researchers to critically consider the historical contexts that shape residential patterning to determine the relevant structural factors in air pollution-health research.

Further, while this work sheds light on the independent effect of RRS on air pollution to demonstrate RRS as a possibly relevant confounder in air pollution-health studies, there remains the critical question about the magnitude of potential confounding by RRS to determine to what extent this bias can impact established associations. Future studies formally quantifying this bias (e.g., quantitative bias analysis) can build on our findings to better understand the effect of confounding by RRS on air pollution-health associations.

We also found that individual race and ethnicity and NSES remain significantly associated with air pollution levels in fully adjusted models. This indicates that, beyond RRS, there are additional social factors that also shape air pollution exposure and should continue to be considered in air pollution-health studies. Our findings with respect to race are consistent with findings from Woo et al,^55^ who demonstrated that racial and ethnic minority individuals are exposed to greater concentrations of air pollutants even after accounting for their neighborhood composition. Since race itself is a poor measure of racism, the variable ends up capturing several forms of structural racism (e.g., interpersonal and institutional) not accounted for by RRS. Therefore, our significant findings could be indicative of both racial discrimination at the individual level (e.g., discrimination by real estate agents)^28,31,74^ as well as at other levels (e.g., labor force, credit markets)^39,75^ that we could not explicitly measure but are also important in shaping racial disparities in air pollution exposure beyond the influence RRS. This aligns with contemporary understanding of structural racism as a multilevel and multidimensional construct that persistently drives race-based inequities across health and its determinants.^76^

To our knowledge, this study is the first to provide estimates for NSES from models that simultaneously adjust for RRS when examining air pollution as an outcome. Other studies have found significant associations between NSES and various health outcomes in models that also include RRS,^77,78^ suggesting that NSES may have an independent effect in the spatial patterning of health and its determinants, including air quality. While the relationship between RRS and NSES is complex, particularly in terms of establishing temporality between the two neighborhood factors, each factor appears to capture different neighborhood effects. This emphasizes the importance of adjustment for both in air pollution studies to avoid potential residual confounding.

Across all social factors, we observed associations of greater magnitude between these factors and NO_2_ compared with PM_2.5_. Variations in the spatial distribution of the two pollutants likely underscore the differences in the magnitude of effects observed in our analysis. NO_2_ is an indicator of traffic-related air pollution that accumulates near its source and has high within-area heterogeneity.^79–81^ PM_2.5_, on the other hand, is considered a long-range pollutant that exhibits less within-area variability.^79^ As such, social factors associated with the opportunity for individuals to live away from polluting sources likely will have a greater impact on an individual’s exposure to a near-source pollutant, such as NO_2_, compared with a long-range source, such as particulate matter.

### Limitations and Strengths

There are important limitations to our study to consider. First, there remains unaddressed heterogeneity of the RRS-air pollution estimates across cohorts. While pooling estimates using a random-effects estimator relaxes the assumption that cohort-specific models are estimating the same true effect, it does not address the heterogeneity. Further analyses (Supplemental Material 6; http://links.lww.com/EE/A323) did not reveal any single predictor driving the underlying heterogeneity to guide appropriate subgroup analyses. As such, the presented estimate of the average true effect may not represent the data well in their totality and, thus, should be interpreted with caution.^82^

Additionally, we operationalize segregation using the Divergence index, a relatively new multirace measure that captures the evenness dimension of residential segregation. This measure was selected due to its decomposable properties, allowing for segregation measurement at finer geographic scales. However, because it is a multirace measure, we were not able to differentiate which racial ethnic group is over- or underrepresented within a given neighborhood. Since a concentration of some racial and ethnic groups may confer more advantages than the concentration of others, we expect that effect estimates for RRS will vary accordingly when using race-specific segregation measures (e.g., isolation). Specifically, we believe that findings from our study likely underestimate the effect of the segregation of racially and ethnically minoritized groups on air pollution burden.^83^ Relatedly, divergence is one of many segregation measures available to measure the spatial separation of racial groups. Results presented in this analysis may vary if alternative segregation measures are used. We encourage researchers to explore RRS-ambient air pollution associations using other measures (e.g., isolation, dissimilarity) to determine if the magnitude and direction of the associations found in this analysis are robust across measures and dimensions of segregation.

Further, exposures were compiled at the Census tract-level because of the geographic scale at which administrative data used to develop the racial segregation and NSES indices was available. It is possible that findings may vary if this analysis were repeated at a different geographic scale.^84^ Future studies should examine the environmental implications of RRS at various geographic scales (e.g., block-level and distance buffers) to compare the magnitude and direction of effects at different scales of measurement. Relatedly, our outcome was defined as residential exposure to air pollution, which may not accurately reflect the totality of an individual’s air pollution exposure.^85^ While we believe residential exposure still has important public health implications, future studies may consider measuring air pollution exposure using an approach that better captures the diversity of environmental contexts individuals experience on a daily basis.

Additionally, all statistical models in this analysis included thin plate splines as spatial terms in an attempt to minimize spatial confounding. There is evidence that shows that the inclusion of spatial terms in models where covariates and the outcome share the same scale of spatial variability can result in biased effect estimates.^86,87^ In our analysis, estimated air pollution surfaces were smoothed using universal kriging, and RRS surfaces were developed using inverse-distance weighting. While the approaches for smoothing slightly differ, it is possible that smoothing in each case may have induced similarities in the spatial structures between the exposure and outcomes. This may have led to the arbitrary apportionment of effects between the covariates and the spatial terms in the model, thereby biasing estimated coefficients. With the differences in smoothing approaches, the variation in the spatial patterning in PM_2.5_ and NO_2_ as long-range and near-source pollutants, respectively, and our uncertainty to how this relates to the scale of variability in RRS, we are unsure to what extent smoothing-induced bias serves to threaten our reported estimates. We encourage future work to explore spatial confounding more thoroughly in RRS-air pollution research, such as employing spatial approaches such as Dupont et al’s^87^ Spatial+ to test the robustness of these findings following better control of spatial confounding.

Further, there is a minor temporal mismatch between neighborhood-level exposures and air pollution measurements for participants’ whose closest observation to 2000 was in 1999. We elected to include these participants to maximize the sample size for all cohorts; however, this means there are some individuals whose air pollution measurement was collected 1 year before their assigned neighborhood exposure. Since we did not expect the neighborhood-level exposures and air pollutant measurements to vary significantly within a year, we chose to include these participants. Nonetheless, this mismatch should be considered in the interpretation of these results. Finally, this analysis is limited to urban participants only. Patterns of RRS in rural areas are more complex,^40,88^ and may result in associations with air pollution that may differ in magnitude and direction than those observed in this analysis. Future work should explore this line of research further.

Despite these limitations, this study has several strengths. First, findings were pooled from several large cohorts with wide geographic representation, conferring more robust estimates and increased generalizability. This is important given the novelty of the research question and the potential for this work to shape future research. Additionally, the use of individual georeferenced data from these cohorts allowed for adjustment of both individual and area-level covariates, and the use of multilevel models to account for both levels simultaneously. Finally, our analysis uses a spatial RRS measurement – an innovation to nonspatial segregation measures that are traditionally used in segregation research in public health, despite the acknowledgment of segregation as an inherent spatial process.^44,43,89^ Developing a spatial segregation measure that accounts for the demographic composition of neighboring communities improves measurement and, in part, overcomes challenges of attempting to measure a phenomenon that does not conform to administrative boundaries.

## Conclusion

Our findings indicate that racial residential segregation, a spatial manifestation of structural racism, is associated with the differential distribution of poor air quality through mechanisms independent of income or individual race and ethnicity. The implications of our findings offer evidence that ongoing efforts to isolate the effects of air pollution on health in air pollution epidemiology studies may be insufficient until they have appropriately accounted for structural underpinnings that drive environmental and health inequities.

## Conflicts of interest statement

The authors declare that they have no conflicts of interest with regard to the content of this report.

## Acknowledgments

We thank the study participants as well as the staff and investigators of each cohort for their valuable contributions.

## Supplementary Material


