# Supplementary material for: Racial residential segregation is associated with ambient air pollution exposure after adjustment for multilevel sociodemographic factors: Evidence from eight US-based cohorts
Source: Environ Epidemiol. 2025 Jan 20;9(1):e367. doi: 10.1097/EE9.0000000000000367 (PMC11749741; doi:10.1097/EE9.0000000000000367)
Supplement: Supplementary file 1 [file ee9-9-e367-s001.pdf]

Supplementary material

Supplemental Material 1: Directed Acyclic Graph illustrating simplified relationships between ambient air pollution and cardiovascular disease mortality

Supplemental Material 2: CONSORT diagram

Supplemental Material 3. Harmonized covariates across cohorts.

Supplemental Material 4. Additional details regarding construction of neighborhood socioeconomic status index.

Supplemental Material 5: RRS distribution of cohort participants included in analytic sample.

Supplemental Material 6. Additional analyses to explore underlying heterogeneity.

Supplemental Material 1: Directed Acyclic Graph illustrating simplified relationships between ambient air pollution and cardiovascular disease mortality

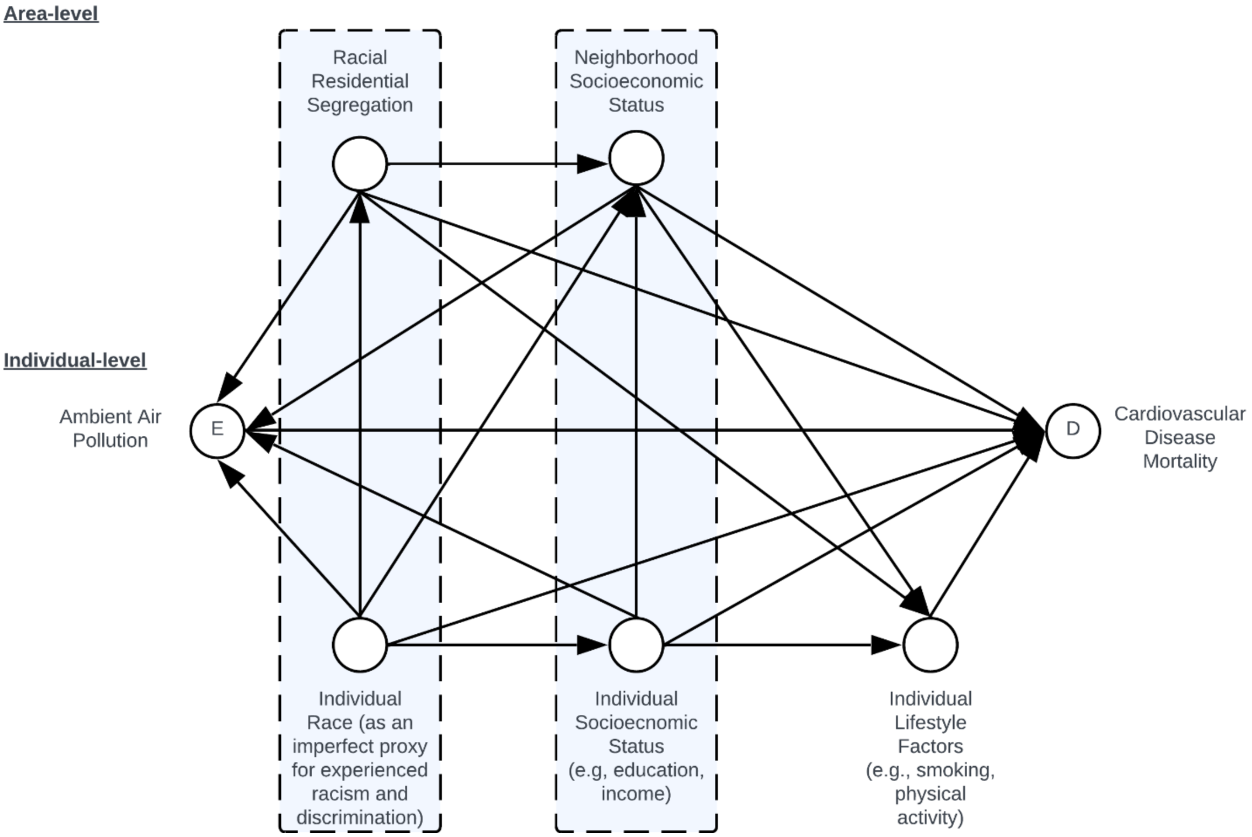

The above figure depicts a simplified Directed Acyclic Graph (DAG) for the relationship between ambient air pollution and cardiovascular disease mortality, as an example health outcome. We include commonly adjusted covariates in air pollution epidemiology research, including neighborhood socioeconomic status, individual race, individual socioeconomic status, and individual lifestyle factors. We also include our conceptualization of racial residential segregation as a potentially relevant confounder in this relationship. We depict RRS and NSES in shaded boxes to demonstrate them as area-level compositional measures made up of the underlying distribution of individuals race and socioeconomic status, respectively. We include individual race in this DAG as a social construct and imperfect proxy for individual experiences of racism, and depict its relationships with other variables as such. For example, the arrow connecting individual race and ambient air pollution is meant to represent instances of individualized racism such as racial discrimination in mortgage lending that can result in shaping the residential air quality a racialized individual is exposed to. Furthermore, please see the discussion for more details on the complex relationship between RRS and NSES which will further explain the direction of the arrow from RRS to NSES. This DAG is not meant to be comprehensive, it is solely meant to communicate how our study team conceptualizes racial residential segregation as a potential confounder on the air pollution → health causal pathway.

Supplemental Material 2: CONSORT diagram

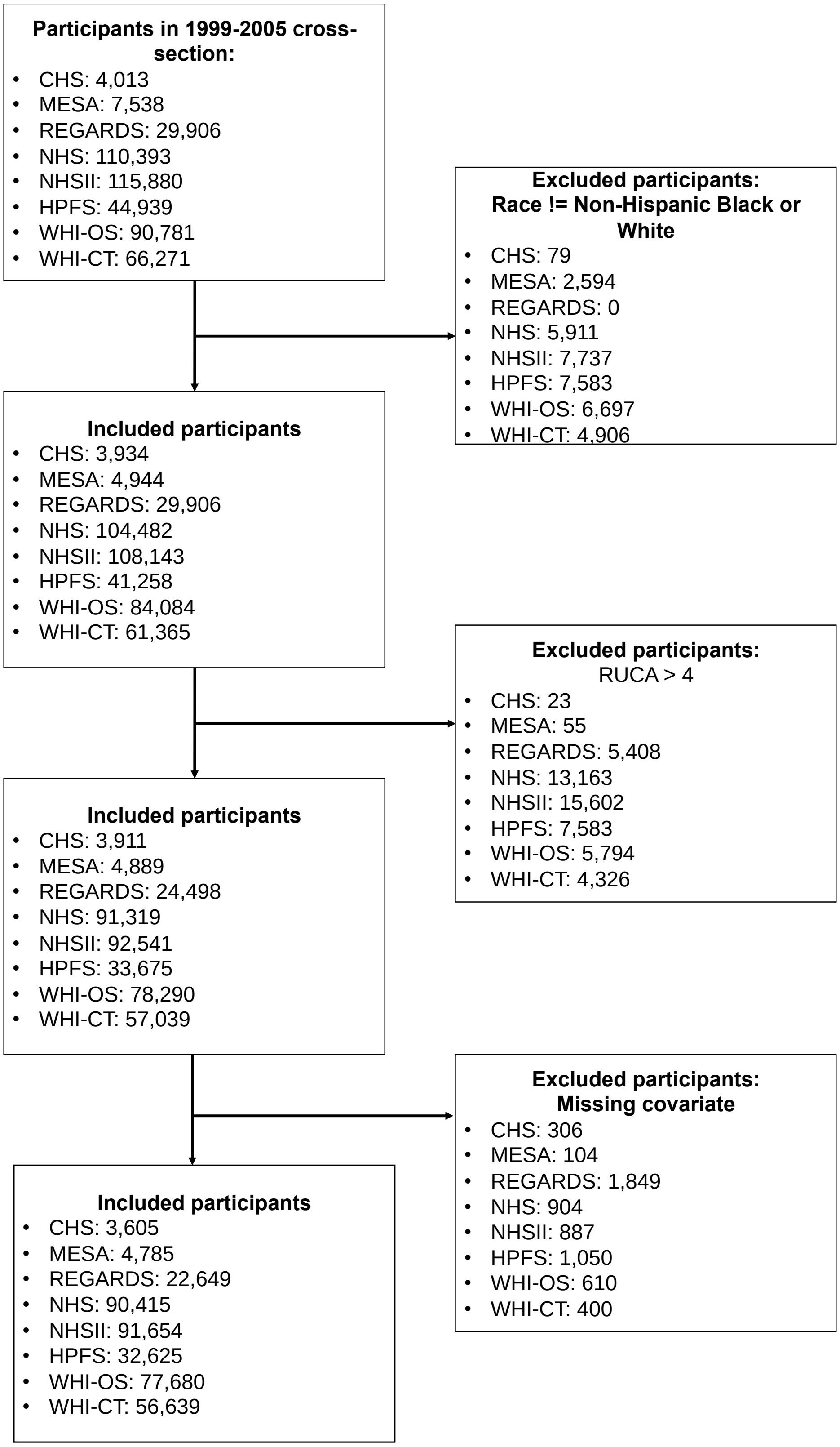

Supplemental Material 3: Table 1. Harmonized covariates across cohorts

	Education		Race and Ethnicity		Sex	
	Cohort-specific	Harmonized	Cohort-specific	Harmonized	Cohort-specific	Harmonized
CHS	0 = NO SCHOOLING<br>1 = GRADE 1<br>2 = GRADE 2<br>3 = GRADE 3<br>4 = GRADE 4<br>5 = GRADE 5<br>6 = GRADE 6<br>7 = GRADE 7<br>8 = GRADE 8<br>9 = GRADE 9<br>10 = GRADE 10<br>11 = GRADE 11<br>12 = GRADE 12<br>13 = GED<br>14 = 1 YEAR VOCATIONAL SCHOOL<br>15 = 2 YEARS VOCATIONAL SCHOOL<br>16 = 3 YEARS VOCATIONAL SCHOOL<br>17 = 1 YEAR COLLEGE<br>18 = 2 YEARS	**<HS** = ("NO SCHOOLING", "GRADE 1", "GRADE 2", "GRADE 3", "GRADE 4", "GRADE 5", "GRADE 6", "GRADE 7", "GRADE 8", "GRADE 9", "GRADE 10", "GRADE 11")<br><br>**HS+** = ("GRADE 12", "GED", 1 YEAR VOCATIONAL SCHOOL”, "2 YEARS VOCATIONAL SCHOOL", "3 YEARS VOCATIONAL SCHOOL", "1 YEAR COLLEGE","2 YE "3 YEARS COLLEGE",	**Race**:<br>1 = White<br>2 = Black<br>3 = Asian/Pacific Islander<br>4 = American Indian/Alaskan Native<br>5 = Other<br>**Hispanic:**<br>0 = No<br>1 = Yes	**Non-Hispanic White** = (Race = “White” & Hispanic = “No”)<br><br>**Non-Hispanic Black =** (Race = “Black” & Hispanic = “No”)<br><br>**Asian/Pacific Islander =** (Race = “Asian/Pacific Islander” & Hispanic = “No”)<br><br>**Native American = (**Race = **“**American Indian/Alaskan Native” & Hispanic = “No”)<br><br>**Other =** (Race = **“**Other ” & Hispanic = “No”)<br>**Hispanic =**	0 = Female<br>1 = Male	**Female** = “Female”<br>**Male** = “Male”

	COLLEGE<br>19 = 3 YEARS COLLEGE<br>20 = 4 YEARS COLLEGE<br>21 = GRADUATE OR PROFESSIONAL	"4 YEARS COLLEGE", "GRADUATE OR PROFESSIONAL")		(Hispanic = "Yes")		
MESA	0 = NO SCHOOLING,<br>1 = GRADES 1-8,<br>2 = GRADES 9-11<br>3 = COMPLETED HIGH SCHOOL/GED<br>4 = SOME COLLEGE BUT NO DEGREE<br>5 = TECHNICAL SCHOOL CERTIFICATE<br>6 = ASSOCIATE DEGREE<br>7 = BACHELOR'S DEGREE<br>8 = GRADUATE OR PROFESSIONAL SCHOOL	**<HS** = ("NO SCHOOLING", "GRADES 1-8", "GRADES 9-11")<br><br>**HS+** = ("COMPLETED HIGH SCHOOL/GED", "SOME COLLEGE BUT NO DEGREE", "TECHNICAL SCHOOL CERTIFICATE", "ASSOCIATE DEGREE", "BACHELOR'S DEGREE", "GRADUATE OR PROFESSIONAL SCHOOL")	**Race**:<br>1 = WHITE, CAUCASIAN<br>2 = CHINESE-AMERICAN<br>3 = BLACK, AFRICAN-AMERICAN<br>4 = HISPANIC/ LATINO	**Non-Hispanic White** = ("WHITE, CAUCASIAN")<br><br>**Non-Hispanic Black** = ("BLACK, AFRICAN-AMERICAN")<br><br>**Asian/Pacific Islander** = ("CHINESE-AMERICAN")<br><br>**Native** = NA<br><br>**Other** = NA<br><br>**Hispanic** = ("HISPANIC/ LATINO ")	0 = Female<br>1 = Male	**Female** = "Female"<br>**Male** = "Male"
REGARDS	1 = <HS<br>2 = HS graduate or	**<HS** = ("<HS")	**Race:**<br>W = White	**Non-Hispanic White** =	F = Female<br>M = Male	**Female** = "Female"

	GED<br>3 = Some college (Some technical college, Technical school grad, some college)<br>4 = College graduate and above (post grad or professional degree"	**HS + =** (“Some college”, “College graduate and above”)	B = Black	(“White”)<br><br>**Non-Hispanic Black** = (“Black”)<br><br>**Asian/Pacific Islander** = NA<br><br>**Native American** = NA<br><br>**Other** = NA<br><br>**Hispanic** = 0		**Male** = “Male”
NHS	(Binary)<br>Doctor<br>Master’s<br>Registered nurse<br>Bachelor’s	**<HS =** NA<br><br>**HS+ = (“**Doctor”, “Master’s”, “Registered nurse”, “Bachelor’s”)	**Race:**<br>1 = White<br>2 = Black<br>3 = American Indian<br>4 = Asian<br>5 = Hawaiian<br>6 = Other/unknown<br>7 = Multi-racial<br><br>**Ethnicity:**<br>1 = Hispanic or Latino<br>2 = Not Hispanic or Latino	**Non-Hispanic White** = (Race = “White” & Ethnicity = “Not Hispanic or Latino”)<br><br>**Non-Hispanic Black =** (Race = “Black” & Ethnicity = “Not Hispanic or Latino”)<br><br>**Asian/Pacific Islander =** (Race = “Asian” | “Hawaiian” & Ethnicity = “Not	0 = Female<br>1 = Male	**Female** = “Female”<br>**Male** = “Male”

				Hispanic or Latino”)<br><br>**Native American** = (Race **=** **“**American Indian & Ethnicity = “Not Hispanic or Latino”)<br><br>**Other** = (Race = **“**Other /Unknown” | “Multi-racial” & Ethnicity = “Not Hispanic or Latino”)<br><br>**Hispanic =** (Ethnicity = “Hispanic or Latino**”**)		
NHSII	Not available - all participants had at least an associate's degree	**<HS** = NA<br><br>**HS + =** All participants	**Race:**<br>1 = White<br>2 = Black<br>3 = American Indian<br>4 = Asian<br>5 = Hawaiian<br>6 = Other/unknown<br>7 = Multi-racial	**Non-Hispanic White** = (Race = “White” & Ethnicity = “Not Hispanic or Latino”)<br><br>**Non-Hispanic Black =** (Race = “Black” &	0 = Female<br>1 = Male	**Female** = “Female”<br>**Male** = “Male”

			**Ethnicity:**<br>1 = Hispanic or Latino<br>2 = Not Hispanic or Latino	Ethnicity = “Not Hispanic or Latino”)<br><br>**Asian/Pacific Islander =** (Race = “Asian” | “Hawaiian” & Ethnicity = “Not Hispanic or Latino”)<br><br>**Native American** (Race = **“**American Indian & Ethnicity = “Not Hispanic or Latino”)<br><br>**Other =** (Race = **“**Other /Unknown” | “Multi-racial” & Ethnicity = “Not Hispanic or Latino”)<br><br>**Hispanic =** (Ethnicity = “Hispanic or Latino**”**)		
HPFS	Not available - all	**<HS** = NA	**Race (binary):**	**Non-Hispanic**	0 = Female	**Female** =

	participants have graduate or professional degree	**HS +** = All participants	1 = white<br>2 = black<br>3 = asian<br>4 = other	**White** = (“white”)<br><br>**Non-Hispanic Black** = (“black”<br><br>**Asian/Pacific Islander =** (“Asian”<br><br>**Native American = NA**<br><br>**Other** (“Other”)<br><br>**Hispanic** = NA	1 = Male	“Female”<br>**Male** = “Male”
WHI-OS	1 = Didn't go to school<br>2 = Grade school<br>3 = Grade school (5-8 years)<br>4 = Some high school (9-11 years)<br>5 = High school diploma of GED<br>6 = Vocational or training school<br>7 = Some college or associate degree<br>8 = College graduate or baccalaureate	**<HS** = (“Didn't go to school”, “Grade school”, “Grade school (5-8 years)”, “Some high school (9-11 years)”)<br><br>**HS + = (“** High school diploma of GED, “Vocational or training school”, “Some college or associate degree”, “College graduate or baccalaureate	**Race =**<br>1 = American Indian/Alaska Native<br>2 = Asian<br>3 = Native Hawaiian/Other PI<br>4 = Black<br>5 = White<br>6 = More than one race<br>9 = Unknown not reported<br><br>**Ethnicity:**	**Non-Hispanic White =** (Race = “White” & Ethnicity = “Not Hispanic/Latino”)<br><br>**Non-Hispanic Black =** (Race = “Black” & Ethnicity = “Not Hispanic/Latino”)<br><br>**Asian/Pacific Islander =** (Race = “Asian” | “Native	1 = Female<br>2 = Male	**Female** = “Female”<br>**Male** = “Male”

	degree<br>9 = Some post-graduate or professional<br>10 = Master's degree<br>11 = Doctoral degree (PhD, MD, JD, etc.)	degree", "Some post-graduate or professional", "Master's degree", "Doctoral degree (PhD, MD, JD, etc.)"	0 = Not Hispanic/Latino<br>1 = Hispanic/Latino<br>9 = Unknown/not reported	Hawaiian/Other PI" & Ethnicity = "Not Hispanic/Latino")<br><br>**Native American =** (Race = "American Indian/Alaska Native" & Ethnicity = "Not Hispanic/Latino")<br><br>**Other =** (Race = "More than one race" | "Unknown not reported & Ethnicity = "Not Hispanic/Latino")<br><br>**Hispanic =** Ethnicity = "Hispanic/Latino"		
WHI-CT	1 = Didn't go to school<br>2 = Grade school<br>3 = Grade school (5-8 years)<br>4 = Some high school (9-11 years)<br>5 = High school diploma of GED<br>6 = Vocational or	**<HS** = ("Didn't go to school", "Grade school", "Grade school (5-8 years)", "Some high school (9-11 years)")<br><br>**HS + =** ("High school diploma of GED,	**Race =**<br>1 = American Indian/Alaska Native<br>2 = Asian<br>3 = Native Hawaiian/Other PI<br>4 = Black<br>5 = White	**Non-Hispanic White =** (Race = "White" & Ethnicity = "Not Hispanic/Latino")<br><br>**Non-Hispanic Black =** (Race = "Black" & Ethnicity = "Not	1 = Female<br>2 = Male	**Female** = "Female"<br>**Male** = "Male"

	training school<br>7 = Some college or associate degree<br>8 = College graduate or baccalaureate degree<br>9 = Some post-graduate or professional<br>10 = Master's degree<br>11 = Doctoral degree (PhD, MD, JD, etc.)	"Vocational or training school", "Some college or associate degree", "College graduate or baccalaureate degree", "Some post-graduate or professional", "Master's degree", "Doctoral degree (PhD, MD, JD, etc.)"	6 = More than one race<br>9 = Unknown not reported<br><br>**Ethnicity:**<br>0 = Not Hispanic/Latino<br>1 = Hispanic/Latino<br>9 = Unknown/not reported	Hispanic/Latino")<br><br>**Asian/Pacific Islander =** (Race = "Asian" | "Native Hawaiian/Other PI" & Ethnicity = "Not Hispanic/Latino")<br><br>**Native American =** (Race = "American Indian/Alaska Native" & Ethnicity = "Not Hispanic/Latino")<br><br>**Other =** (Race = "More than one race" | "Unknown not reported & Ethnicity = "Not Hispanic/Latino")<br><br>**Hispanic =** Ethnicity = "Hispanic/Latino"		

Supplemental Material 4. Additional details regarding construction of neighborhood socioeconomic status index

Census variables, detailed in Supplemental Material Table 1, were compiled from the 2000 Decennial Census. Census variables were selected based on variables used for the MESA neighborhood socioeconomic status index. Included variables were reverse coded as needed so that higher values indicate worse SES, and transformed as needed to have approximately normal distributions. Principal components analysis (PCA) was then used to derive weights using the transformed and reverse coded Census tract estimates. The first component for each year was retained and loadings were used as weights (Supplemental Table 1). Estimates for each census variable were standardized and multiplied by the corresponding weight and then summed to derive the NSES index value for each Census tract. NSES values were mean-centered within-year. Higher index values indicate greater neighborhood disadvantage.
Tracts with <100 total population, <30 housing units, or ≥33% in group quarters were excluded.

Supplemental Material 3: Table 1. Census variables compiled for years 2000

**Domain**	**Variable name**	**Description**	**Reverse Coded**	**Transformed**	**PCA Weights**
Education	bach_rev	Percent of persons 25 or older with at least a Bachelor's degree	Yes	No	0.260
Education	hs_rev	Percent of persons 25 or older with at least high school education	Yes	No	0.289
Employment	unemployed_cbrt	Percent unemployed among civilians 16 and over in the labor force	No	Yes, cube root	0.271
Employment	notlabor	Percent of civilians 16 and over not in the labor force	No	No	0.203
Housing	no_car_cbrt	Percent of housing units without vehicle	No	Yes, cube root	0.259
Housing	med_own_occ_houseval_cbrt	Median value of occupied housing units	No	Yes, cube root	-0.223

Housing	own_occ_housing_rev	Percent of housing units that are owner occupied out of total housing units	Yes	No	0.223
Housing	occ_housing_rev_cbrt	Percent of occupied housing units	Yes	Yes, cube root	0.152
Income/Wealth	hhpubass_cbrt	Percent of households with public assistance	No	Yes, cube root	0.296
Income/Wealth	hhintdivnet_rev	Percent of households with interest, dividends, or net rental income	Yes	No	0.297
Income/Wealth	hhinc50kplus_rev	Percent households with household income>$50,000	Yes	No	0.308
Income/Wealth	med_hhinc_cbrt	Median household income	No	Yes, cube root	-0.325
Income/Wealth	totpov_cbrt	Percent of persons below the poverty level	No	Yes, cube root	0.316
Occupation	mang_prof_rev	Percent with management, professional, and related occupation	Yes	No	0.260
Residential Stability	samehouse_rev_cbrt	Percent living in the same house as one year ago	Yes	Yes, cube root	0.019

Supplemental Material 5. RRS distribution of cohort participants included in analytic sample.

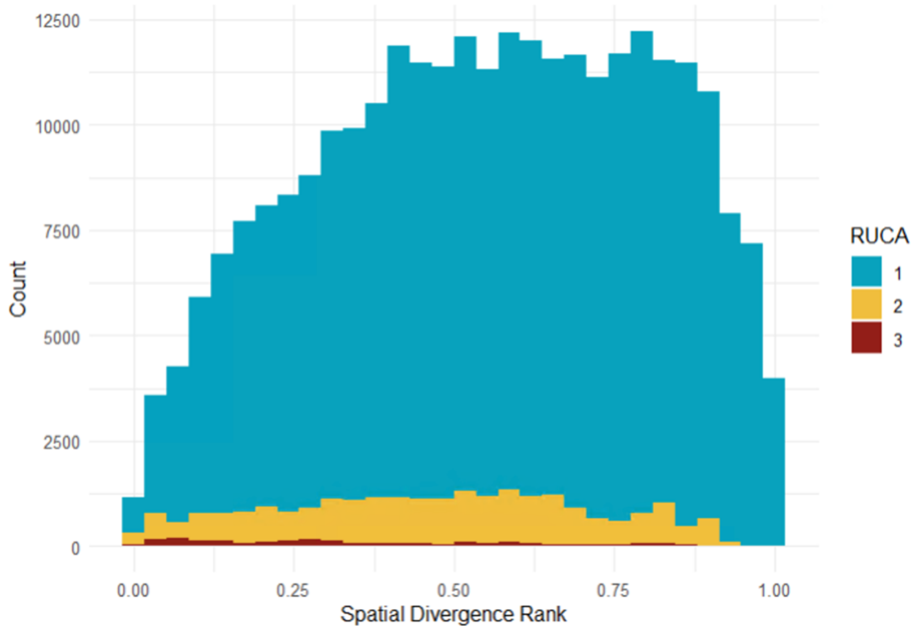

Supplemental Material 6. Additional analyses to explore underlying heterogeneity.

Simple meta-regression was used to explore potential significant predictors of heterogeneity observed in RRS-air pollution random effects meta-analysis models. Results from the meta regression are displayed in the table below (**Supplemental Material 6: Table 1)** and visualized using bubble plots (**Supplemental Material 6: Figure 1)**. We examined median RRS, median NSES, and proportion of tracts with a RUCA value of 1, as predictors. I^2 values, and p-values from Q-tests for residual heterogeneity and Test of Moderators, are presented below. The I^2 value quantifies amount of variability in our data that can be attributed to remaining between-study heterogeneity after accounting for a given predictor. The p-value from Q-tests indicate whether the remaining heterogeneity not explained by the predictor is significant at an alpha level of 0.05. The p-value from the Test of Moderators indicates whether the tested predictor is a significant predictor in the variation of effect sizes across cohorts, also at an alpha level of 0.05. From this additional analysis, we see that no one predictor can sufficiently explain the observed heterogeneity in meta-analysis models. Only degree of urbanicity, measured by the proportion of participants living in tracts with a RUCA score of 1, was a significant predictor of the observed heterogeneity in RRS-NO2 meta-analysis models (Test of Moderators p = 0.06); however, there remained a significant amount of between-study heterogeneity even after accounting for this predictor (I^2 value = 93.5%, Q-test p-value = < 0.001).

Supplemental Material 6: Table 1. Tests of heterogeneity results

**Predictor**	**I^2 value (%)**	**Q-tests (*p*-value)**	**Test of Moderators (*p*-value)**
RRS-$PM_{2.5}$			
Median RRS (rank)	86.0	< 0.001	0.468
Median NSES (z-score)	80.3	< 0.001	0.073
Proportion in RUCA = 1	84.8	< 0.001	0.288
RRS-NO_2			
Median RRS (rank)	96.6	< 0.001	0.788
Median NSES (z-score)	96.0	< 0.001	0.279
Proportion in RUCA = 1	93.5	< 0.001	0.016

Supplemental Material 6: Figure 1. Bubble plot to visualize meta regression results.

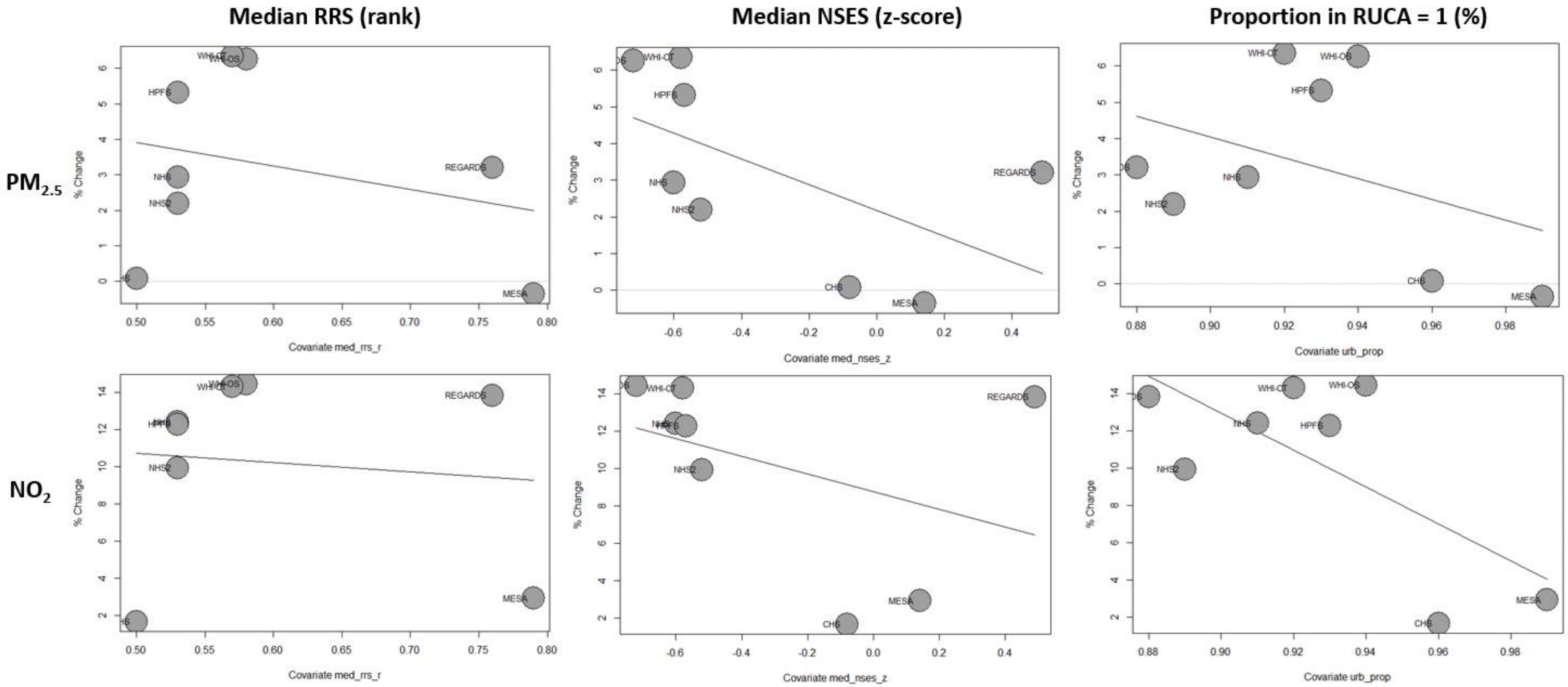

Bubble plots were used to visualize meta-regression results. Effect size (for the association between RRS an $PM_{2.5}$ or NO_2) values are on the y-axis and values for the respective predictor (i.e., median RRS rank, Median NSES z-score, Proportion in RUCA = 1) are on the x-axis. Each cohort (n=8) is plotted based on the estimated effect size and value for the respective predictor.
